# Dynamic Behavior of Poly(*N*-isopropylmethacrylamide) in Neat Water and in Water/Methanol Mixtures

**DOI:** 10.1021/acs.langmuir.4c01515

**Published:** 2024-07-09

**Authors:** Chia-Hsin Ko, Patrick Wastian, Dirk Schanzenbach, Peter Müller-Buschbaum, André Laschewsky, Christine M. Papadakis

**Affiliations:** † TUM School of Natural Sciences, Physics Department, Soft Matter Physics Group, Technical University of Munich, James-Franck-Straße 1, 85748 Garching, Germany; ‡ Institut für Chemie, Universität Potsdam, Karl-Liebknecht-Straße 24-25, 14476 Potsdam-Golm, Germany; § TUM School of Natural Sciences, Physics Department, Chair for Functional Materials, Technical University of Munich, James-Franck-Straße 1, 85748 Garching, Germany; ∥ Fraunhofer-Institut für Angewandte Polymerforschung, Geiselbergstraße 69, 14476 Potsdam-Golm, Germany

## Abstract

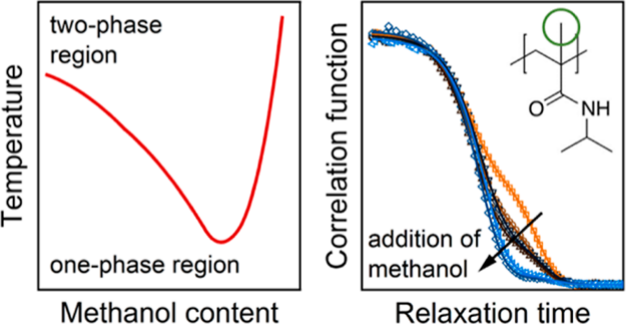

We investigate the collective dynamics of thermoresponsive polymer poly(*N*-isopropylmethacrylamide) (PNIPMAM) in aqueous solution and in water/methanol mixtures in the one-phase region. In neat water, the polymer concentration *c* is varied in a wide range around the overlap concentration c*, that is estimated at 23 g L^–1^. Using dynamic light scattering (DLS), two decays (“modes”) are consistently observed in the intensity autocorrelation functions for *c* = 2–150 g L^–1^ with relaxation rates which are proportional to the square of the momentum transfer. Below c*, these are attributed to the diffusion of single chains and to clusters from PNIPMAM that are formed due to hydrophobic interactions. Above c*, they are assigned to the diffusion of the chain segments between overlap points and to long-range concentration fluctuations. From the temperature-dependent behavior of the overall scattering intensities and the dynamic correlation lengths of the fast mode, the critical temperatures and the scaling exponents are determined. The latter are significantly lower than the static values predicted by mean-field theory, which may be related to the presence of the large-scale inhomogeneities. The effect of the cosolvent methanol on the dynamics is investigated for polymer solutions having *c* = 30 g L^–1^ and methanol volume fractions in the solvent mixtures of up to 60 vol %. The phase diagram was established by differential scanning calorimetry. The slow mode detected by DLS becomes significantly weaker as methanol is added, i.e., the solutions become more homogeneous. Beyond the minimum of the coexistence line, which is located at 40–50 vol % of methanol, the dynamics is qualitatively different from the one at lower methanol contents. Thus, going from the water-rich to the methanol-rich side of the miscibility gap, the change of interaction of the PNIPMAM chains with the two solvents has a severe effect on the collective dynamics.

## 1 Introduction

Thermoresponsive polymers with lower critical solution temperature (LCST) behavior in aqueous solution have been proposed, among others, for biomedical and for sensor and switching applications.^1−6^ In spite of the fact that these applications require different kinds of thermoresponsive polymers, most fundamental investigations have focused on poly(*N*-isopropylacrylamide) (PNIPAM).^7^ Its cloud point, *T*_cp_, in H_2_O is about 31 °C in a wide range of molar masses and concentrations;^7^ thus, it belongs to the type II class of LCST polymers.^8^ Its macroscopic phase behavior as well as its switching mechanisms and time scales were found to be strongly related to its interactions with water, i.e., the formation of hydrogen bonds by the amide group and the hydrophobic hydration of the backbone and the isopropyl group.^9−13^ Recently, the dynamics of the water molecules forming the hydration shell of the hydrophobic groups was identified as an important factor for the behavior of PNIPAM in aqueous solution.^14,15^

Poly(*N*-isopropylmethacrylamide) (PNIPMAM) is very similar to PNIPAM, except that it features an additional α-methyl group on the backbone. A number of studies have addressed the its phase behavior^16−25^ and its molecular interactions with water.^15,24,26−32^ It emerges that PNIPMAM is thermoresponsive with LCST behavior of type II in aqueous solution and cloud points between 40 and 50 °C. The higher *T*_cp_ of PNIPMAM compared to the one of PNIPAM^24,25^ was explained by the fact that, below *T*_cp_, the hydrophobic groups of PNIPMAM are more hydrated than the ones of PNIPAM,^24^ while the amide groups of PNIPMAM are less hydrated than the ones of PNIPAM.^30^ Moreover, in PNIPMAM, the intermolecular interactions between the amide groups were found to be much weaker than the ones in PNIPAM, which was tentatively attributed to steric hindrances.^30^ The higher degree of hydration in PNIPMAM below *T*_cp_ was confirmed in atomistic simulations.^15^ These also showed that, due to the steric hindrance of the additional α-methyl group, the isopropyl-backbone distance is increased, which hampers the collapse of the chain.^15^ Moreover, PNIPMAM features a lower degree of the hydration of the backbone, but a higher degree of hydration of the isopropyl group, and a higher residence time of water near the chain than PNIPAM.^15^ Hence, the effect of the α-methyl group on the behavior of PNIPMAM in aqueous solution is complex and deserves further investigation.

Previously, we carried out structural studies of aqueous PNIPMAM solutions in a wide concentration range (polymer concentration *c* = 2 to 150 g L^–1^).^23,24^ Using turbidimetry, we established the phase diagram and found that the cloud point temperature *T*_cp_ decreases slightly from 45 to 42 °C with increasing concentration. Using small-angle neutron scattering (SANS) on solutions having *c* = 30–150 g L^–1^, the chain conformation of PNIPMAM in the one-phase state was found to be more compact than the one of PNIPAM.^24^ Loosely packed, large-scale inhomogeneities (>100 nm) and physical cross-links were detected already in the one-phase state, even for the lowest concentration studied.^24^ It was found that the critical exponents related to the local concentration fluctuations deviate strongly from the mean-field values, which was tentatively attributed to the presence of large-scale inhomogeneities. Raman spectroscopy revealed that, in the one-phase state, the hydrophobic groups of the PNIPMAM chains are more hydrated than the ones of the PNIPAM chains.^24^

Only few studies have addressed the dynamics of PNIPAM or PNIPMAM, which are very sensitive to large-scale inhomogeneities. In dilute aqueous solutions of PNIPAM, dynamic light scattering (DLS) revealed the hydrodynamic radii of the single chain, but no slower dynamic processes were observed.^33−36^ In more concentrated PNIPAM solutions, two diffusive relaxation processes (“modes”) were observed.^37−41^ The fast process is due to the relaxation of chain segments between neighboring overlap points with dynamic correlation lengths of the order of a few nanometers. Hence, this correlation length may be associated with the distance between overlap points. The slow mode has dynamic correlation lengths in the range of a few 100 nm, and it was attributed to long-range concentration fluctuations, which are due to intermolecular interactions between PNIPAM via hydrogen bonds.^38^ In another study on PNIPAM, the two modes were attributed to the diffusion of short chain segments (blobs) and to the relaxation of the cage made by a number of blobs, respectively.^39^ In our DLS investigations on concentrated (9 and 25 wt %) aqueous solution of PNIPAM, the two modes were detected as well.^40,41^ The scaling exponent of the correlation length of the fast mode was found to be ν_dyn_ = 0.70 ± 0.06 (“dyn” refers to dynamic), which is close to the value characteristic of 3D Ising behavior, in consistency with the strong large-scale dynamic inhomogeneities.^41^ To the best of our knowledge, the dynamics of PNIPMAM in aqueous solution has so far only been investigated in an extremely diluted solution, revealing the collapse/swelling of the single chains upon heating/cooling through *T*_cp_.^21^ More concentrated solutions have not been addressed.

In numerous studies, the effect of a cosolvent on PNIPAM in aqueous solution was investigated, most of these addressing the cosolvent methanol in solutions, microgels and thin films of PNIPAM.^40−50^ Upon addition of methanol, *T*_cp_ decreases until a minimum is reached in a range of methanol volume fractions in the solvent mixture, ϕ_m_, typically in the range of 0.2–0.35.^44^ This behavior of *T*_cp_ is termed “co-nonsolvency”, and its origin is still under debate.^50^ At higher ϕ_m_-values, *T*_cp_ increases steeply, and the behavior is dominated by the interactions of the polymer with methanol.^48^ In our previous SANS study on a 3 wt % PNIPAM solution in an 80:20 v/v water/methanol mixture, we identified (static) critical exponents related to the local concentration fluctuations which differ strongly from the values predicted by mean-field theory.^47^ Our DLS experiments revealed that the slow mode is also present in concentrated PNIPAM solutions in water/methanol mixtures (ϕ_m_ = 0.10–0.20).^40,41^ The scaling exponents in neat water and in water/methanol were found to differ from each other.^41^ The phase diagram of PNIPMAM in water/methanol mixtures was recently established^25^ and shows qualitatively similar behavior to the one of PNIPAM, only with higher *T*_cp_ values throughout (molar fractions of methanol in the solvent up to 0.4 were investigated) and the minimum of the coexistence line being shifted to lower methanol fractions. Time-resolved investigations on a PNIPMAM thin film in a mixed water/methanol vapor showed that the release of water from PNIPMAM is markedly slower than the uptake of methanol.^51^ Hence, PNIPMAM differs in many ways from PNIPAM, namely regarding the chain conformation, the intermolecular interactions with water and a cosolvent, the transition behavior and the interplay between water and methanol on the chain.

Here, we use DLS to investigate the collective dynamics of PNIPMAM in aqueous solution in the one-phase state and in a wide range of polymer concentrations (2–150 g L^–1^), i.e., across the overlap concentration. The results from temperature-dependent measurements allow us to determine the critical exponent of the scattered intensity, γ, and of the dynamic correlation lengths of the local concentration fluctuation, ν_dyn_, in dependence on polymer concentration. Furthermore, the effect of the cosolvent methanol on the dynamics is investigated in a wide range of methanol volume fractions (ϕ_m_ = 0–0.6) for a semidilute PNIPMAM solution (*c* = 30 g L^–1^).

## 2 Materials and Methods

### 2.1 Materials

**Figure 1:**
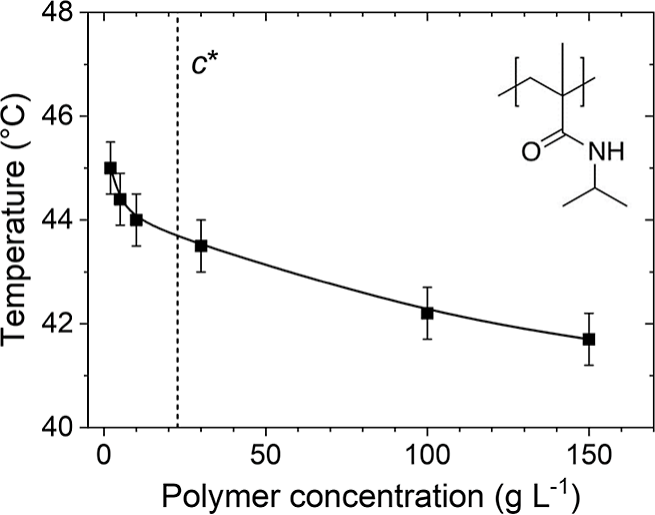
Phase diagram of PNIPMAM in D_2_O. Squares: cloud points *T*_cp_ determined using turbidimetry, data taken from ref (24) The line guides the eye. The vertical dashed line indicates the overlap concentration c*. The chemical structure of the repeating unit of PNIPMAM is shown in the inset.

PNIPMAM having an apparent number-average molar mass  = 17 000 g mol^–1^ and a dispersity *D̵* = 1.74 was synthesized by conventional free radical polymerization.^24^ The chemical structure of the repeat unit is shown in the inset of Figure 1 below. The overlap concentration c* of PNIPMAM in aqueous solution may be estimated by the relation^52^
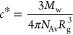
1where *M*_w_ is the weight-average molar mass (29 600 g mol^–1^), *N*_Av_ Avogadro’s constant and *R*_g_ the radius of gyration. For an extremely dilute aqueous PNIPMAM solution below *T*_cp_, a ratio *R*_g_/*R*_h_ = 1.6 was determined.^21^ Using this value together with the *R*_h_ value of the sample studied here, *R*_h_ = 4.2 nm, determined from the correlation length of the fast mode at 25 °C and *c* = 5 g L^–1^ (see below), results in *R*_g_ = 6.7 nm and thus in an estimate for c* of 23 g L^–1^.

As solvents, D_2_O (99.9 atom % D) from Sigma-Aldrich and fully deuterated methanol (CD_3_OD, ≥99.8 atom % D, water impurities ≤0.025%) were used. Samples having polymer concentrations *c* = 2–150 g L^–1^ and volume fractions of CD_3_OD in the solvent mixture, ϕ_m_ = 0–0.6, were prepared by adding the solvents to the preweighed polymer and shaking the solutions for prolonged time at room temperature. The solutions were filtered using filters with a pore size of 0.2 μm.

### 2.2 Methods

Differential scanning calorimetry (DSC) measurements were conducted in the same way as in our previous study.^24^ In brief, a DSC 3 STARe system from Mettler Toledo was used. The measurements were carried out in a temperature range of 10–80 °C. Each sample first underwent a heating and cooling cycle to erase the thermal history. The onset and the peak temperatures, *T*_onset_ and *T*_peak_, were determined from the second heating scan, carried out at a rate of 1 K min^–1^. The enthalpies of the phase transition were obtained from the area under the endothermic peak, and the thermograms and the enthalpies were normalized to the mass of the polymer solution, *g*_s_.

Refractometry was carried out using an Abbe refractometer from A. Krüss Optronic. The sample temperature was controlled using a F32-HE circulator (JULABO Labortechnik GmbH, Seelbach, Germany). Temperature-dependent refractive indices of the polymer solutions, *n*, were measured for neat D_2_O and for PNIPMAM solutions having concentrations of 30–150 g L^–1^ up to the respective *T*_cp_. For concentrations of 2–10 g L^–1^, the values were interpolated. The refractive indices of 30 g L^–1^ PNIPMAM solutions in D_2_O/CD_3_OD mixtures with ϕ_m_ = 0.1–0.6 were measured up to the respective cloud point. The concentration- and temperature-dependent refractive indices *n* are given in Figure S1 in the Supporting Information.

Viscometry was carried out on the D_2_O/CD_3_OD solvent mixtures having ϕ_m_ = 0–0.6. An AMVn Automated Micro Viscometer from Anton Paar and the software RheoPlus for AMV were used. For each sample, 10 measurements were carried out, and the results were averaged. The such obtained viscosities η along with fits of the Vogel–Fulcher–Tammann law are given in Figure S2 in the Supporting Information.

DLS was carried out using an instrument with an ALV-5000/E correlator (ALV-Laser Vertriebsgesellschaft mbH, Langen, Germany), an ALV/SO-SIPD photomultiplier, to which the signal was fed by an optical fiber, a HeNe laser having a power of 35 mW and a wavelength λ = 632.8 nm, and a goniometer. The solutions were filled into cylindrical cuvettes, that were mounted in an index-matching vat, which was filled with toluene and was thermostated by a JULABO F32 thermostat (JULABO Labortechnik GmbH, Seelbach, Germany). Angle-dependent measurements were carried out at temperatures between 20 and 25 °C at scattering angles θ = 30–135° in steps of 15–25°. At each angle, 3–10 measurements of a duration of 20–30 s were performed. Temperature-dependent measurements were carried out at a scattering angle θ = 90° between 15 and 25 °C and the respective cloud point *T*_cp_. At each temperature, 10 measurements having a duration of 20–30 s were carried out. A waiting time of 10 min was applied after each temperature change. In some cases, several heating runs were carried out, and no difference between different heating runs was observed.

The autocorrelation functions were analyzed using the REPES algorithm,^53,54^ which is implemented in the Gendist software and calculates the distribution functions of relaxation times τ, *A*(τ). These are given in equal area representation, τ *A*(τ) vs log(τ), and feature two peaks for all samples, reflecting a fast and a slow mode. After discarding outliers, the resulting areas of the peaks, *A*_fast_ and *A*_slow_, as well the mean relaxation times of the two peaks in the distribution functions, τ_fast_ and τ_slow_, were obtained by averaging the values from the 3–10 measurements. When several heating runs were carried out, the results were averaged.

The area fraction of the slow peak, *f*_slow_, was calculated as follows
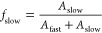
2

The relaxation rates of both modes, Γ_i_, where i stands for fast or slow, were in all cases linearly dependent on the squared momentum transfer, *q*^2^, as verified in angle-dependent measurements. Using Fick’s second law of diffusion
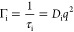
3where Γ_i_ is the relaxation rate and *q* the momentum transfer, namely
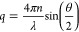
4the diffusion coefficients *D*_fast_ and *D*_slow_ were calculated from the Γ_i_- and the *q*-values at θ = 90°, using the values of the refractive index *n* of the particular PNIPMAM solution (Figure S1 in the Supporting Information). The dynamic correlation lengths ξ_fast_ and ξ_slow_ were determined by the Stokes–Einstein relation
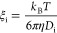
5where η is the temperature-dependent viscosity of the solvent (Figure S2 in the Supporting Information).

## 3 Results and Discussion

We discuss first the dynamics of PNIPMAM in neat water for polymer concentrations in a wide range across the overlap concentration. Then, we turn to the changes of the dynamics in mixtures of water and methanol for a PNIPMAM concentration above the overlap concentration. The deuterated solvents D_2_O and CD_3_OD were chosen for comparison with our previous results from neutron scattering.^24^

### 3.1 Dynamics in Neat Water in Dependence on Polymer Concentration and Temperature

As a guideline for our investigations of the concentration and temperature dependence of the dynamics in aqueous PNIPMAM solutions in D_2_O, we briefly revisit the phase diagram of this system (Figure 1):^24^ The cloud point *T*_cp_ from turbidimetry (onset of the decay of the light transmission measured at a wavelength of 632.8 nm and a heating rate of 0.2 K min^–1^) decreases from 45.0 °C at a polymer concentration *c* = 2 g L^–1^ to 41.8 °C at 150 g L^–1^. DLS experiments address solutions in this concentration range and in the temperature range between 15 °C and the respective *T*_cp_-value.

**Figure 2:**
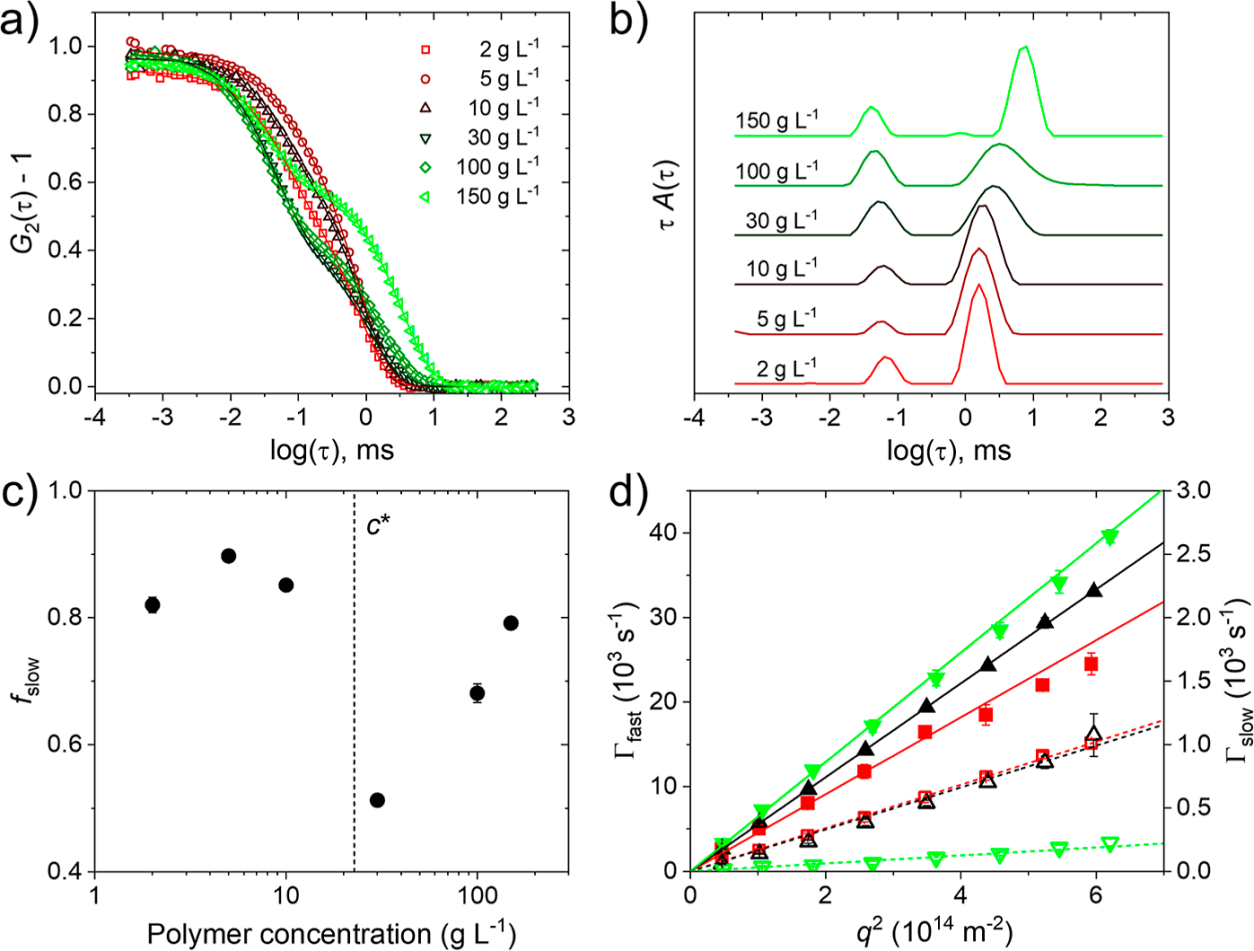
(a) Autocorrelation functions from DLS on the PNIPMAM solutions in D_2_O at 25 °C at θ = 90° (symbols). For clarity, only every second experimental data point is shown. The lines are the fits corresponding to the distributions of relaxation times shown in (b). The distribution functions were normalized to the height of the peak at ca. 0.03–0.1 ms, and they were shifted vertically by equal amounts. (c) Relative amplitude of the slow mode, *f*_slow_, in dependence on polymer concentration *c*. The vertical dashed line indicates the estimated overlap concentration c*. (d) Relaxation rates Γ_fast_ (closed symbols, left axis) and Γ_slow_ (open symbols, right axis) in dependence on *q*^2^ for *c* = 5 (red squares), 30 (black triangles up) and 150 g L^–1^ (green triangles down). The lines are linear fits through the origin.

The DLS intensity autocorrelation functions of PNIPMAM solutions in neat D_2_O having polymer concentrations between 2 and 150 g L^–1^, all measured at 25 °C, i.e., far below the respective *T*_cp_, and a scattering angle θ = 90° consistently show two decays (Figure 2a), and the corresponding distributions of relaxation times show two peaks, which are located at 0.01–0.1 ms and above 1 ms, respectively (Figure 2b). The area fraction of the slow peak, *f*_slow_, shows nonmonotonous behavior (eq 2, Figure 2c). Interestingly, it is rather high (∼0.8–0.9) up to the estimated overlap concentration c* ≅ 23 g L^–1^ (see the Materials and Methods section). In the dilute regime, we attribute the slow mode to small clusters consisting of several PNIPMAM chains, which possibly appear due to hydrophobic interchain interactions. As the concentration is increased across c*, *f*_slow_ decreases to ∼0.5 at *c* = 30 g L^–1^ and then increases steadily to 0.8 at 150 g L^–1^. In this regime, we attribute *f*_slow_ to long-range concentration fluctuations of the semidilute polymer concentration, that become more pronounced with increasing polymer concentration. We note that the peaks in the distribution function related to the slow process become broader, as c* is crossed, i.e., there is a qualitative change of behavior.

**Figure 3:**
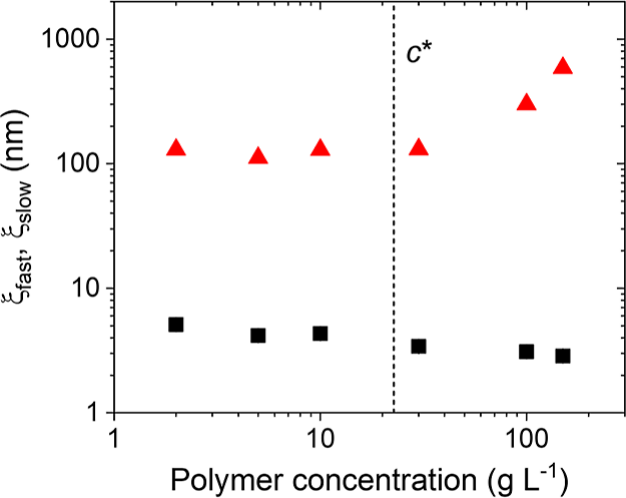
Dynamic correlation lengths ξ_fast_ (black squares) and ξ_slow_ (red triangles) at 25 °C in dependence on PNIPMAM concentration in neat D_2_O on a double-logarithmic scale. The vertical dashed line indicates c*.

The nature of the two modes emerges from angle-dependent measurements. The relaxation rates of both modes, Γ_i_, where *i* stands for “fast” or “slow”, depend linearly on the squared momentum transfer, *q*^2^, as shown exemplarily for *c* = 5, 30, and 150 g L^–1^ in Figure 2d. This dependency enables us to calculate the dynamic correlations lengths ξ_fast_ and ξ_slow_ (eqs 3–5, Figure 3).

At *c* = 2–10 g L^–1^, i.e., below c*, the correlation lengths are ξ_fast_ = 4.2–5.1 nm and ξ_slow_ ≅ 120–130 nm. In this concentration regime, we attribute ξ_fast_ to the hydrodynamic radius of the single chains. The presence of the slow mode and thus dynamic inhomogeneities even far below c* are unexpected and different from the findings on dilute aqueous solutions of PNIPAM.^33−36^ Thus, they may be attributed to clusters formed by means of hydrophobic interactions between the isopropyl groups in the side groups and the methyl groups on the backbone.

Above c*, ξ_fast_ decreases slightly from 3.4 nm at 30 g L^–1^ to 2.9 nm at 150 g L^–1^, i.e., the distance between overlap points decreases with increasing polymer concentration. The behavior is similar to the one of the static correlation length from SANS observed by us previously.^24^ ξ_slow_ increases strongly with concentration from 130 nm at 30 g L^–1^ to 590 nm at 150 g L^–1^, i.e., the long-range concentration fluctuations grow significantly with increasing polymer concentration. Moreover, they become more and more pronounced, as seen from the increase of the relative amplitude of the slow mode with concentration (Figure 2c). Our previous SANS experiments on solutions having *c* = 30–150 g L^–1^, i.e., above c*, revealed forward scattering from large inhomogeneities below *T*_cp_ as well.^23,24^ This underlines that the slow mode is due to clusters of PNIPMAM chains, that are connected by hydrophobic interaction.

**Figure 4:**
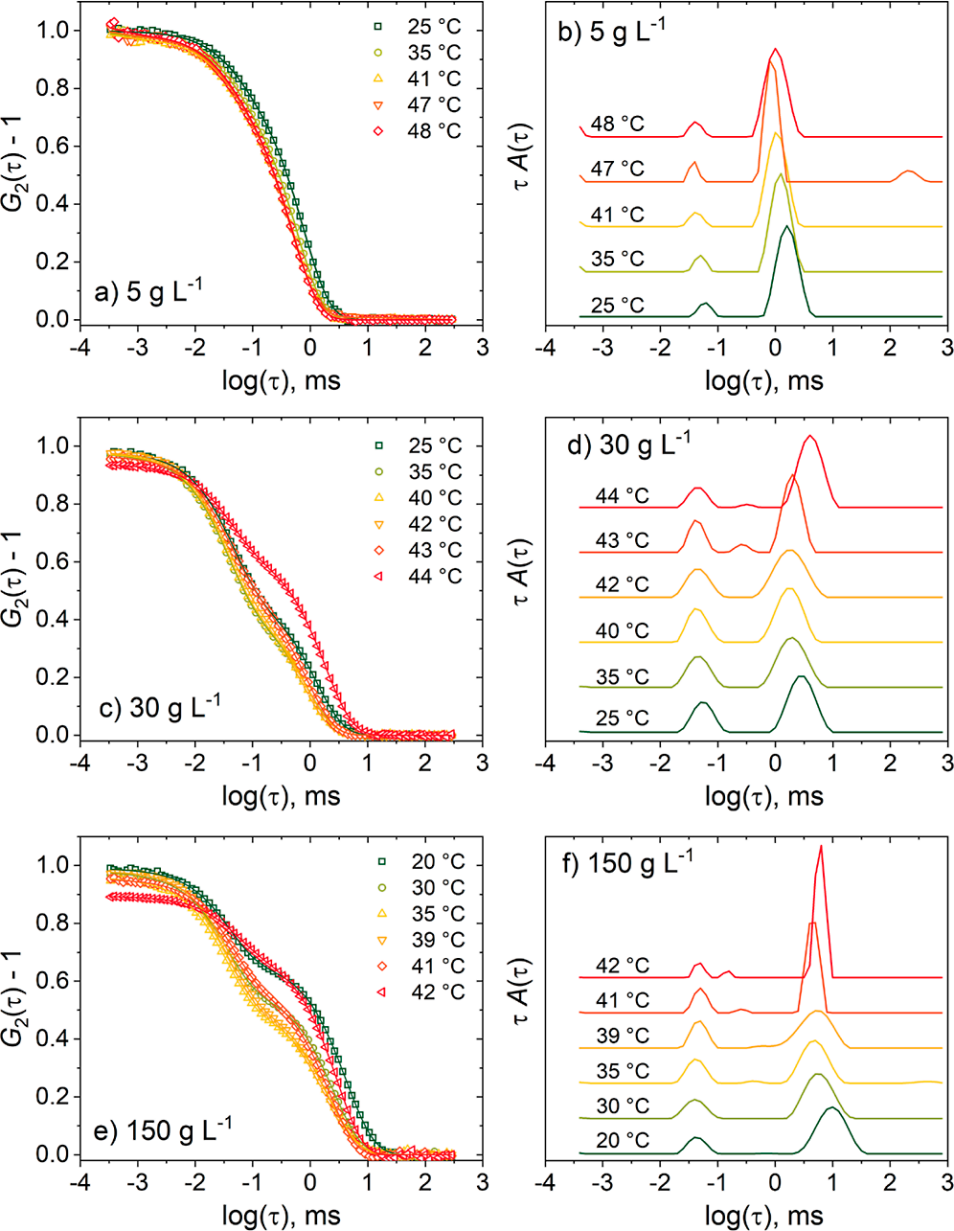
Representative DLS data of the PNIPMAM solutions in neat D_2_O from heating runs for the polymer concentrations of 5 g L^–1^ (a, b), 30 g L^–1^ (c, d) and 150 g L^–1^ (e, f). Left panel: Autocorrelation functions at θ = 90° and the temperatures given in the legends. For clarity, only every second experimental data point is shown (symbols). The lines are the fits corresponding to the distributions of relaxation times shown in the right panel. The distribution functions are shifted vertically by equal amounts.

To characterize the temperature-dependent behavior upon approaching the respective cloud point, *T*_cp_, all solutions were measured in dependence on temperature at a scattering angle θ = 90°. Representative autocorrelation functions and the corresponding distribution functions are shown in Figure 4. Far below *T*_cp_, they show only slight variations in dependence on temperature, whereas, as *T*_cp_ is approached, the slow decay becomes more prominent, especially for *c* = 30 g L^–1^ and above. Above *T*_cp_, the intensity fluctuations were too strong, preventing the correlator from calculating proper autocorrelation functions.

**Figure 5:**
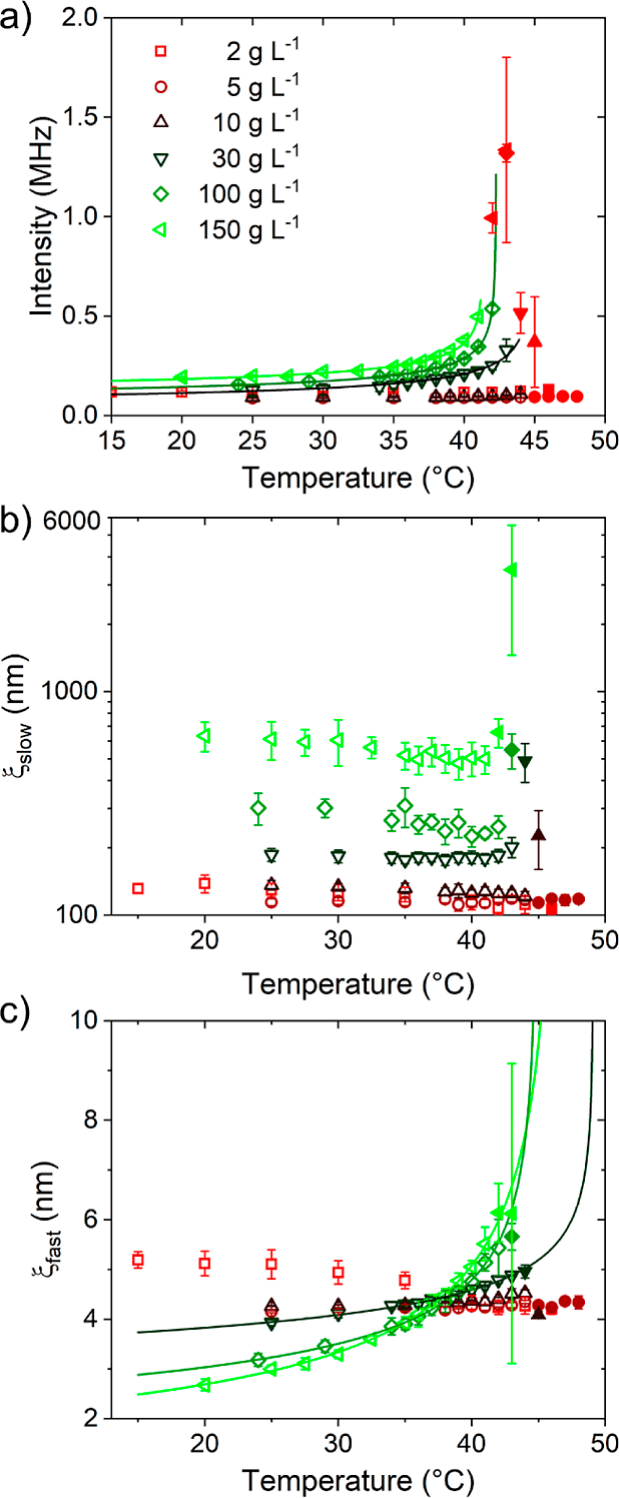
Results from temperature-dependent DLS measurements on PNIPMAM solutions in neat D_2_O at the concentrations given in (a). Open symbols: below *T*_cp_, closed symbols: above. (a) Overall scattered intensities in dependence on temperature (symbols). The lines are fits of eq 6. (b) Correlation lengths of the slow mode, ξ_slow_ in a semilogarithmic representation (b), and the fast mode, ξ_fast_ (c). The lines in (c) are fits of eq 7.

The overall scattering intensities are plotted as a function of temperature in Figure 5a. It is seen that they are lowest and nearly temperature-independent for *c* = 2–10 g L^–1^, i.e., below c*. At *c* = 30–150 g L^–1^, the intensities are higher and increase strongly, as *T*_cp_ is approached. In this concentration range, the temperature dependence up to *T*_cp_ can be described by
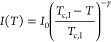
6*I*_0_ is a constant, *T*_c,I_ the critical temperature of the intensity and γ the critical exponent of the intensity. The fits describe the data well (Figures 5a and S3a in the Supporting Information) and result in the *T*_c,I_-values given in Table 1. For 100 and 150 g L^–1^, these are equal to the *T*_cp_-values, whereas for 30 g L^–1^, *T*_c,I_ is slightly higher than *T*_cp_. γ decreases from 0.38 at 30 g L^–1^ to 0.25 at 150 g L^–1^ (Table 1). These values are significantly lower than the mean-field prediction, γ = 1. It is interesting that the deviation from the mean-field value increases with polymer concentration, which may be due to the increasing importance of long-range fluctuations, i.e., the higher relative amplitude of the slow mode (Figure 4c,e). The values are significantly lower than the ones determined previously by us by SANS on 100 and 150 g L^–1^ solutions (γ = 0.64 ± 0.06 and 0.78 ± 0.10). This may be due to the fact that, here, the overall scattering intensity is considered, whereas the exponent determined by SANS related to the single chain scattering only.^24^

**Table 1:** Results from the PNIPMAM Solutions in Neat D_2_O in Dependence on Polymer Concentration *c*^a^

*c* (g L^–1^)	*T*_cp_ (°C)	γ	*T*_c,I_ (°C)	ν_dyn_	*T*_c,ξ_ (°C)
30	43.5 ± 0.5	0.38 ± 0.06	45 ± 1	0.16 ± 0.02	49.1 ± 0.8
100	42.3 ± 0.5	0.30 ± 0.01	42.28 ± 0.04	0.28 ± 0.02	45.0 ± 0.5
150	41.7 ± 0.5	0.25 ± 0.02	41.4 ± 0.2	0.46 ± 0.04	47 ± 1

a Cloud points *T*_cp_, critical exponents γ and critical temperatures, *T*_c,I_, derived from the scattering intensity, dynamic critical exponents, ν_dyn_, and critical temperatures, *T*_c,ξ_, of the correlation length of the fast mode.

**Figure 6:**
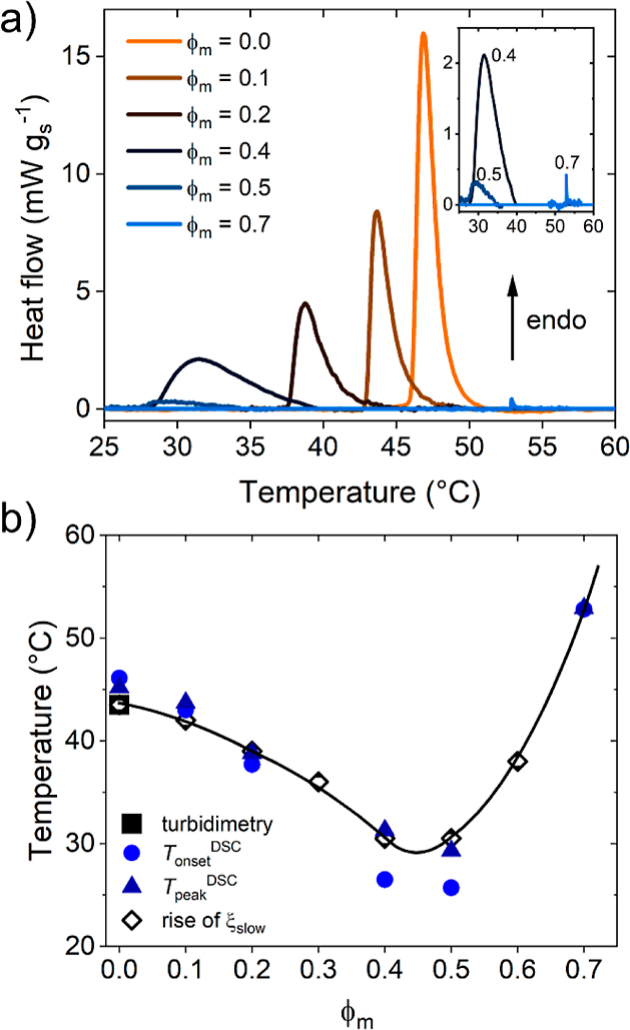
(a) DSC thermograms of the 30 g L^–1^ PNIPMAM solutions in D_2_O/CD_3_OD for the volume fractions of CD_3_OD, ϕ_m_, given in the legend. The inset is a close-up of the thermograms for the ϕ_m_ values given in the graph. (b) Cloud points from turbidimetry and onset temperature *T*_onset_ and peak temperature *T*_peak_ from DSC, as indicated. The transition temperatures determined from the sudden rise of ξ_slow_ are given as well. The line guides the eye.

The correlation length, ξ_slow_, that is calculated from τ_slow_ using eqs 3–5 along with the temperature-dependent solvent viscosities, is given in Figure 5b. For *c* = 2–10 g L^–1^, it is in the range of 100–140 nm and does not show any concentration dependence. We attribute this length scale to the size of the clusters. At 30–150 g L^–1^, ξ_slow_ is higher, namely 180–600 nm, decreases slightly with temperature and increases steeply in a range of a few Kelvin below *T*_cp_. The nonmonotonous behavior of ξ_slow_ in this concentration range indicates counteracting effects, which may be due to the variation of the interaction of the chains with water and with each other, i.e., hydrogen bonding and hydrophobic interactions. We note that the temperature where the sudden increase of ξ_slow_ occurs, coincides with *T*_cp_ from turbidimetry (see also the data for ϕ_m_ = 0 in Figure 6b below). Hence, the latter criterion seems to be a reliable way of determining *T*_cp_.

The correlation lengths of the fast mode, ξ_fast_, are extracted from τ_fast_ using eqs 3–5 along with the temperature-dependent solvent viscosities and are given in Figure 5c. At concentrations of 2–10 g L^–1^, the correlation lengths are nearly constant and in the range 4–5 nm, and we assign these to the hydrodynamic radii of the single chains. In contrast, at 30–150 g L^–1^, the ξ_fast_ values increase with temperature, which reflects the expected contraction of the chains, as *T*_cp_ is approached. We fitted the following scaling law to the latter data
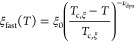
7ξ_0_ is a constant, and *T*_c,ξ_ and ν_dyn_ are the critical temperature and the dynamic critical exponent of ξ_fast_. The temperature dependencies are well described by this expression (Figures 5c and S3b in the Supporting Information). The dynamic exponent ν_dyn_ increases with concentration from 0.16 to 0.46 (Table 1), hence, it approaches the mean-field prediction of the static correlation length, ν = 0.5. However, the critical temperatures *T*_c,ξ_ (Table 1) are far above the *T*_cp_ and *T*_c,I_ values, i.e., the dynamics of the segments between the overlap points do not seem to follow the same behavior as the overall scattered intensity, that also includes the scattering from the long-range concentration fluctuations. The concentration dependences of the exponents γ and ν_dyn_ are contrary to each other, which indicates that at small and large length scales, different interactions may dominate. For 30 g L^–1^, the values of ξ_fast_ are a factor of 2 larger than the static ones identified previously by us by SANS (2 nm, rather independent of temperature), whereas the difference is larger for 100 and 150 g L^–1^: For these concentrations, the values found by SANS are ca. 1.1 and 0.8 nm at 30 °C and increase with temperature up to 2.0 nm at *T*_cp_.^24^ The static exponent ν was found to be 0.34 ± 0.04 and 0.36 ± 0.06 for 100 and 150 g L^–1^,^24^ which is in the same range as the values of ν_dyn_ reported in Table 1. The differences of the length scales and the scaling exponents are in line with previous findings on polymer solutions,^55^ where it was found that additional information on topological constraints is needed to compare results from static and dynamic scattering methods.

### 3.2 Influence of Methanol on the Dynamics

The polymer concentration of 30 g L^–1^ was chosen to investigate the effect of methanol, i.e., a semidilute solution. The phase diagram was determined by DSC. The thermograms measured during slow heating (1 K min^–1^) are shown in Figure 6a. It is seen that the shape of the endothermic peak changes with increasing ϕ_m_: while it is rather symmetric at ϕ_m_ = 0, the high-temperature tail becomes more smeared out, and hence the peak shape becomes more asymmetric, as ϕ_m_ is increased to 0.5. For ϕ_m_ = 0.4 and 0.5, the peaks are very broad. In contrast, at ϕ_m_ = 0.7, the peak is very sharp and weak, i.e., the nature of the transition changes from the water-rich to the methanol-rich side of the phase diagram. The onset and peak temperatures, *T*_onset_ and *T*_peak_, are compiled in Figure 6b. In neat D_2_O and a wide range of polymer concentrations, *T*_onset_ was previously found to be ca. 2 °C higher than *T*_cp_,^24^ which is a small deviation compared to the overall changes upon addition of CD_3_OD. *T*_onset_ (the temperature closest to *T*_cp_) decreases to 26 °C at a volume fraction of methanol ϕ_m_ = 0.5 and increases again strongly to 53 °C at ϕ_m_ = 0.7. This behavior is in agreement with the one reported in ref (25). The enthalpies of the transition are given in Figure S4 in the Supporting Information. They decrease up to ϕ_m_ = 0.5 and stay at a low value at ϕ_m_ = 0.7, which is another hint to the change of the nature of the transition as the minimum of the coexistence line is crossed.

**Figure 7:**
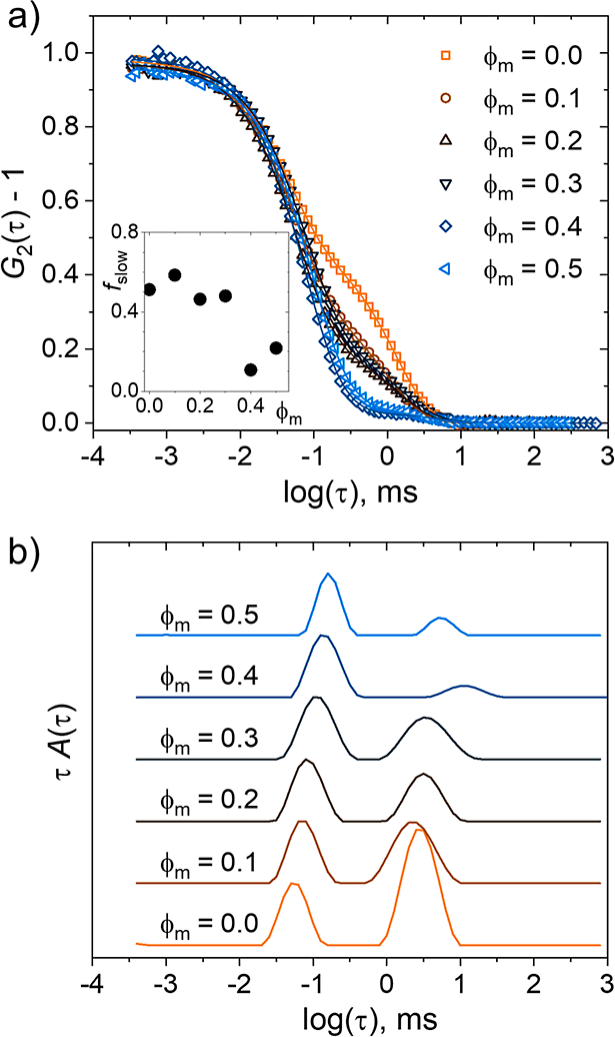
DLS data of the 30 g L^–1^ PNIPMAM solutions in D_2_O/CD_3_OD at 25 °C in dependence on the volume fraction of CD_3_OD, ϕ_m_, as indicated in the graphs. (a) Autocorrelation functions measured at θ = 90°. For clarity, only every second experimental data point is shown (symbols). The lines are the fits corresponding to the distributions of relaxation times shown in (b). The distribution functions were normalized to the height of the peak at ca. 0.01–0.1 ms, and they were shifted vertically by equal amounts. The inset in (a) shows the relative amplitude of slow mode in dependence on polymer concentration *c*.

Figure 7 shows autocorrelation functions and the corresponding distribution functions at a temperature of 25 °C and a scattering angle of 90° for different ϕ_m_-values. The two modes are still observed in the presence of CD_3_OD and move only slightly along the time axis, as ϕ_m_ is increased; however, the area fraction of the slow mode decreases strongly between ϕ_m_ = 0.3 and 0.4 (inset of Figure 7a). This decrease indicates that the solutions become more homogeneous at large length scales and/or that the clusters contain more solvent, and hence, contribute less to the overall scattering. The nature of the two modes stays diffusive (see Figure S5 in the Supporting Information).

**Figure 8:**
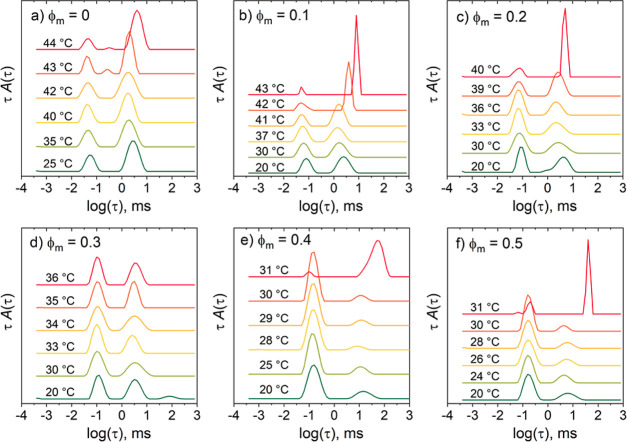
Representative DLS distribution functions from the 30 g L^–1^ PNIPMAM solutions in D_2_O/CD_3_OD mixtures from heating runs for the volume fractions of CD_3_OD, ϕ_m_, and temperatures indicated in the graphs, measured at θ = 90°. The distribution functions are shifted vertically by equal amounts.

**Figure 9:**
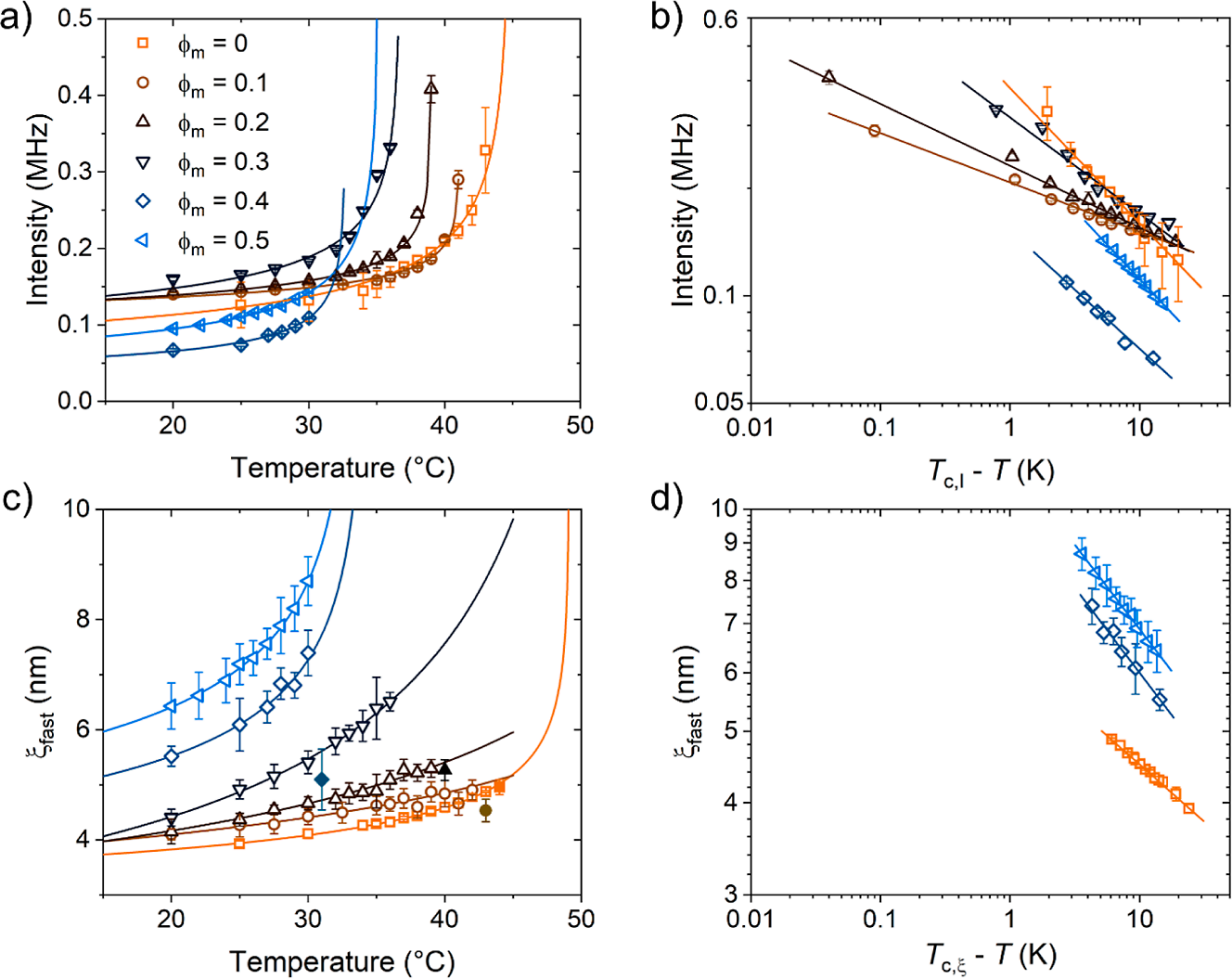
Results from DLS on 30 g L^–1^ PNIPMAM solutions in D_2_O/CD_3_OD mixtures. (a) Overall scattered intensities in dependence on temperature for the volume fractions of CD_3_OD given in the legend (symbols). (b) Same data, plotted in double-logarithmic representation vs *T*_c,I_ – *T*. The lines in (a) and (b) are fits of eq 6 to the data below *T*_cp_. (c) Correlation lengths of the fast mode, ξ_fast_, in dependence on temperature (symbols). (d) Same data at temperatures below *T*_cp_ (symbols), plotted in double-logarithmic representation vs *T*_c,ξ_ – *T*. The lines in (c) and (d) are fits of eq 7 to the data below *T*_cp_. In (a) and (c), open symbols denote values from below *T*_cp_ and closed symbols from above.

**Figure 10:**
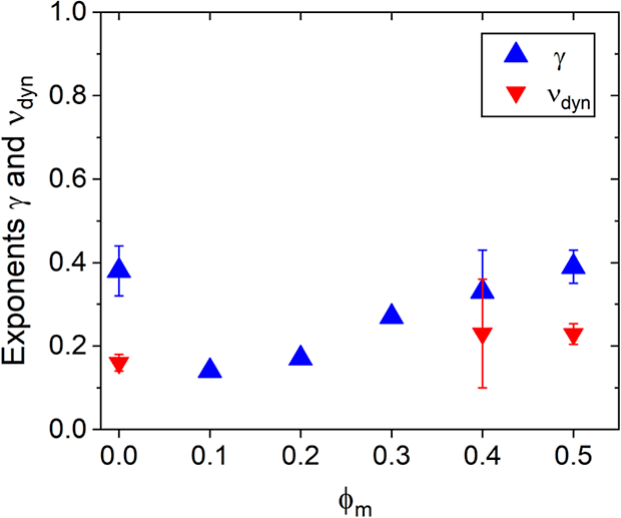
Critical exponents of the intensity, γ (blue triangles up), and the fast correlation length, ν_dyn_ (red triangles down), in dependence on the volume fraction of CD_3_OD in the solvent mixture, ϕ_m_.

**Figure 11:**
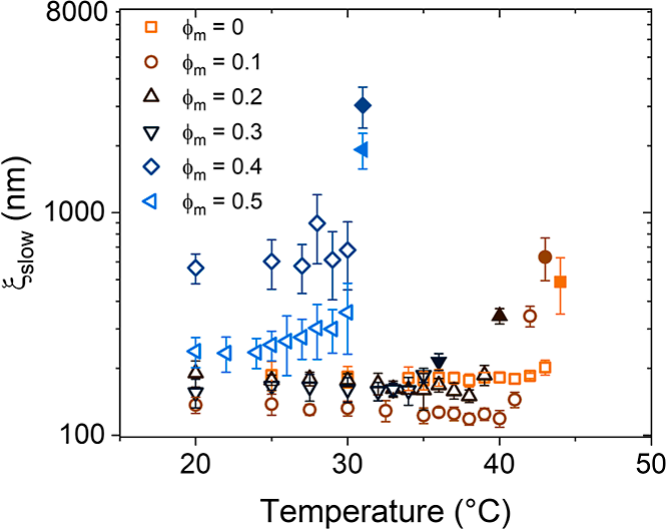
Correlation length of the slow mode, ξ_slow_, in semilogarithmic representation in dependence on temperature for the 30 g L^–1^ PNIPMAM solution in D_2_O/CD_3_OD mixtures for the volume fractions of CD_3_OD given in the graph. Open symbols denote values from below *T*_cp_ and closed symbols from above.

The temperature-dependent autocorrelation functions and distribution functions for ϕ_m_ = 0–0.5 (Figure S6 in the Supporting Information, Figure 8) do not reveal any qualitative changes, as CD_3_OD is introduced, except that, for ϕ_m_ = 0.4, the slow mode is slower than for the other ϕ_m_ values. The temperature-dependent overall intensities follow scaling behavior, as evident from the goodness of the fits of eq 6 (Figure 9a,b). The resulting values of *T*_c,I_ and γ are compiled in Table 2. As expected, *T*_cp_ (determined from the sudden increase of ξ_slow_, see Figure 11 below) and *T*_c,I_ decrease with increasing ϕ_m_, and *T*_c,I_ is similar to *T*_cp_ up to ϕ_m_ = 0.3. However, *T*_c,I_ is ca. 5 °C larger than *T*_cp_ for ϕ_m_ = 0.4 and 0.5. The exponent γ decreases from 0.38 at ϕ_m_ = 0 to 0.14 at ϕ_m_ = 0.1, then increases steadily to 0.39 at ϕ_m_ = 0.5 (Figure 10). The reason for this nonmonotonous behavior is at present unclear. In the entire range, the values are far below the value from mean-field theory.

**Table 2:** Results from the PNIPMAM Solutions of the 30 g L^–1^ PNIPMAM Solutions in D_2_O/CD_3_OD in Dependence on the Volume Fraction of CD_3_OD in the Solvent Mixture, ϕ_m_^a^

ϕ_m_	*T*_cp_ (°C)	γ	*T*_c,I_ (°C)	ν_dyn_	*T*_c,ξ_ (°C)
0	43.5 ± 0.5	0.38 ± 0.06	45 ± 1	0.16 ± 0.02	49.1 ± 0.8
0.1	42.0 ± 0.5^b^	0.14 ± 0.01	41.1 ± 0.1	0.31 ± 0.27^c^	67 ± 32
0.2	39.0 ± 0.5^b^	0.17 ± 0.01	39.0 ± 0.1	0.46 ± 0.31^c^	67 ± 23
0.3	36.0 ± 0.5^b^	0.27 ± 0.01	36.8 ± 0.5	0.62 ± 0.13^c^	55 ± 5
0.4	30.5 ± 0.5^b^	0.33 ± 0.10	35.3 ± 0.8	0.23 ± 0.13	34 ± 5
0.5	30.5 ± 0.5^b^	0.39 ± 0.03	35.3 ± 0.8	0.23 ± 0.03	33.6 ± 0.7

a Cloud points *T*_cp_, critical exponents γ and critical temperatures, *T*_c,I_, derived from the scattering intensity, dynamic critical exponents, ν_dyn_, and critical temperatures, *T*_c,ξ_, of the correlation length of the fast mode.

b From the steep rise in ξ_slow_, see Figure 11.

c Values arguable because of the high value of *T*_c,ξ_.

The behavior of ξ_fast_ with ϕ_m_ is quite complex: In the entire temperature range, ξ_fast_ increases with ϕ_m_ (Figure 9c) and can formally be described by eq 7. However, for ϕ_m_ = 0.1–0.3, very high values are obtained for *T*_c,ξ_ (Table 2). The reason for this behavior is unclear, and we do not consider the related exponents further. For the other ϕ_m_ values, ν_dyn_ scatters around 0.2, i.e., a very low value (Table 2 and Figure 10).

The correlation length of the slow mode, ξ_slow_, below *T*_cp_ is in the range of 100–800 nm for ϕ_m_ = 0–0.5 (Figure 11). For ϕ_m_ = 0–0.3, the values are temperature-independent and increase only in a narrow temperature range below the respective *T*_cp_. At ϕ_m_ = 0.4 and 0.5, the values are significantly higher, already far below the respective *T*_cp_. Interestingly, for ϕ_m_ = 0.5, ξ_slow_ is smaller than for ϕ_m_ = 0.4, and its value increases with temperature. This distinct behavior may indicate a change of behavior from the water-dominated to the methanol-dominated behavior. The steep increase of ξ_slow_ seen at a certain temperature is attributed to the crossing of *T*_cp_ (Table 2). We note that *T*_cp_ decreases strongly between ϕ_m_ = 0.3 and 0.4 (Figure 6b). In this range, also the behavior of the overall intensity as well as ξ_fast_ and ξ_slow_ changes qualitatively. At ϕ_m_ = 0.4 and 0.5, the values of *T*_cp_ are similar to each other, and the expected minimum of the curve may lie in this range.

**Figure 12:**
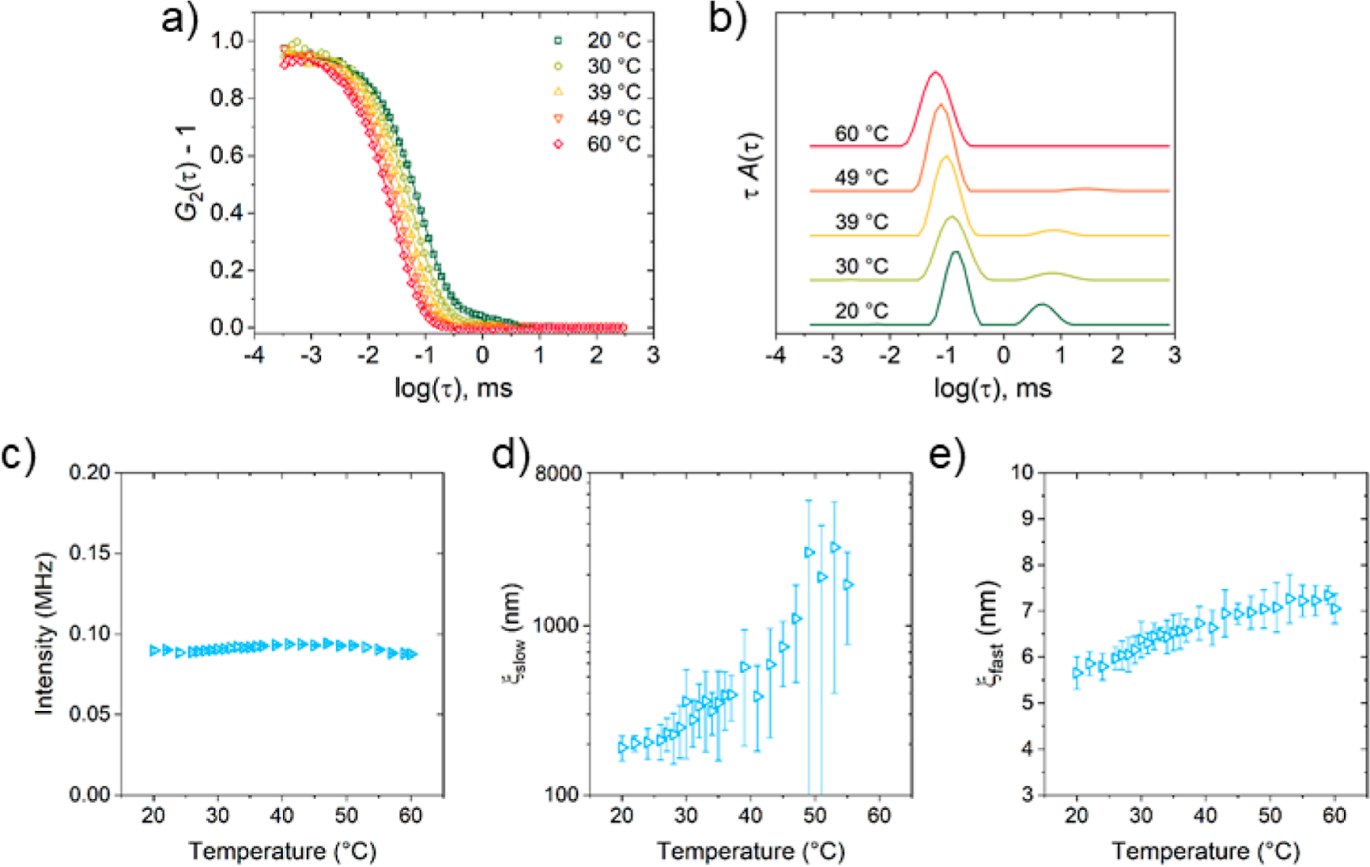
DLS data of the 30 g L^–1^ PNIPMAM solution in a D_2_O/CD_3_OD mixture having ϕ_m_ = 0.6 at 25 °C. (a) Autocorrelation functions measured at θ = 90°. For clarity, only every second experimental data point is shown (symbols). The lines are the fits corresponding to the distributions of relaxation times shown in (b). The distribution functions were shifted vertically by equal amounts. (c) Overall scattered intensity in dependence on temperature. (d) Correlation length of the slow mode, ξ_slow_, in semilogarithmic representation and (e) correlation length of the fast mode, ξ_fast_, in dependence on temperature.

To verify whether the transition to the methanol-dominated behavior has indeed been reached, the behavior of a 30 g L^–1^ PNIPMAM solution in a D_2_O/CD_3_OD mixture with ϕ_m_ = 0.6 is characterized in the same way (Figure 12). The two modes are still observed and are diffusive (Figure S5f in the Supporting Information), however, the slow mode is very weak (Figure 12a,b). The overall scattered intensity is rather constant over the entire temperature range (20–60 °C) with a slight decrease above 47 °C (Figure 12c). ξ_slow_ increases from ca. 400 nm at 37 °C to 570 nm at 39 °C (Figure 12d). At higher temperatures, the relative amplitude of the slow mode is very low, resulting in a high scatter of the values of ξ_slow_, but they seem to increase further to a few 1000 nm. These large aggregates presumably precipitate, which results in the observed decrease of the intensity (Figure 12c). ξ_fast_ increases steadily from 5.7 nm at 20 °C to 6.9 nm at 43 °C and then levels off. This behavior is very different from the one at ϕ_m_ up to 0.5, and eqs 6 and 7 do not describe the temperature dependencies of the scattered intensity and ξ_fast_. We conclude that the coexistence line increases steeply between ϕ_m_ = 0.5 and 0.6, and possibly, there is a transition at ϕ_m_ = 0.6 and 38 °C, which would be consistent with the onset temperature from DSC at ϕ_m_ = 0.7 (Figure 6b).

## 4 Conclusions

We investigated the collective dynamics of the thermoresponsive polymer poly(*N*-isopropylmethacrylamide) (PNIPMAM) in water and in water/methanol mixtures. In neat D_2_O, DLS consistently showed two modes, even at the lowest PNIPMAM concentration of 2 g L^–1^, and at temperatures far below the respective *T*_cp_. The finding of a slow mode, assigned to clusters of PNIPMAM having a size of ca. 100 nm, even in the one-phase state, in addition to the diffusion of single chains, is contradictory to the previous findings from computer simulations and from infrared and Raman spectroscopy that the hydrophobic groups of PNIPMAM are more hydrated than the ones of PNIPAM.^15,24,30^ Thus, the interactions between PNIPMAM chains seem to be more complex and deserve further study.

Above the overlap concentration, the two modes are assigned to the dynamics of the chain segments between overlap points and large-scale inhomogeneities, respectively. Scaling behavior is identified for the overall scattered intensity and the dynamic correlation length of the fast mode. Deviations from our previous results from static SANS studies and from mean-field predictions are present, which require further information, e.g. on topological constraints of the system,^55^ which have not yet been reported. We note that, in semidilute solutions of a high molar mass polystyrene in cyclohexane, the distribution of relaxation times of the slow mode was found to be significantly broader than in the system studied here.^55^ It was speculated that the slow mode is related to the viscoelastic properties of the solutions.^55^

Upon addition of methanol (CD_3_OD), the phase behavior is qualitatively similar to the one of PNIPAM: the coexistence curve, as determined by differential scanning calorimetry, shows a minimum and, subsequently, a steep rise, and the characteristics of the endothermic peaks change qualitatively from the water-rich to the methanol-rich regime. On the water-rich side, i.e., up to ϕ_m_ = 0.5, scaling behavior is observed for the overall intensity as well as for the fast dynamic correlation length; however, the behavior of the scaling exponents is nonmonotonous. The slow mode weakens for ϕ_m_-values above 0.3, and at ϕ_m_ = 0.6, the overall intensity as well as the fast dynamic correlation lengths do not exhibit scaling behavior as in the water-rich region, i.e., the behavior of this solution is fundamentally different.

Overall, we conclude that the DLS experiments provide a wealth of information that may serve as a link between the findings of the local scale (e.g., the interaction of the solvents with the different groups in the side chain and the backbone and the distance between the side chains) and the macroscopic scale, i.e., the phase behavior and the switching behavior.

## Supporting Information Available

Refractive indices of the polymer solutions. Viscosities of the solvent mixtures. Additional results from DLS and DSC (PDF)
