# Supplementary material for: Dynamic Behavior of Poly(N-isopropylmethacrylamide) in Neat Water and in Water/Methanol Mixtures
Source: Langmuir. 2024 Jul 9;40(29):15150–60. doi: 10.1021/acs.langmuir.4c01515 (PMC11270994; doi:10.1021/acs.langmuir.4c01515)
Supplement: Supplementary file 1 — la4c01515_si_001.pdf [file la4c01515_si_001.pdf]

Supporting Information for Dynamic Behavior of Poly(*N*-isopropylmethacrylamide) in Neat Water and in Water/Methanol Mixtures

Chia-Hsin Ko[1], Patrick Wastian[1], Dirk Schanzenbach[2],

Peter Müller-Buschbaum[3], André Laschewsky[2,4], Christine M. Papadakis[1,]*

[1]Technical University of Munich, TUM School of Natural Sciences, Physics Department, Soft Matter Physics Group, James-Franck-Straße 1, 85748 Garching, Germany

[2]Institut für Chemie, Universität Potsdam, Karl-Liebknecht-Straße 24-25, 14476 Potsdam-Golm, Germany

[3]Technical University of Munich, TUM School of Natural Sciences, Physics Department, Chair for Functional Materials, James-Franck-Straße 1, 85748 Garching, Germany

[4]Fraunhofer-Institut für Angewandte Polymerforschung, Geiselbergstraße 69, 14476 Potsdam-Golm, Germany

S1. Refractive Indices of the Polymer Solutions

Figure S1 shows the temperature-dependent refractive indices of the polymer solutions at different polymer concentrations *c* in D_2O (Figure S1a) and the ones at a polymer concentration *c* = 30 g L^{-1} in D_2O/CD_3OD mixtures of different compositions (Figure S1b).

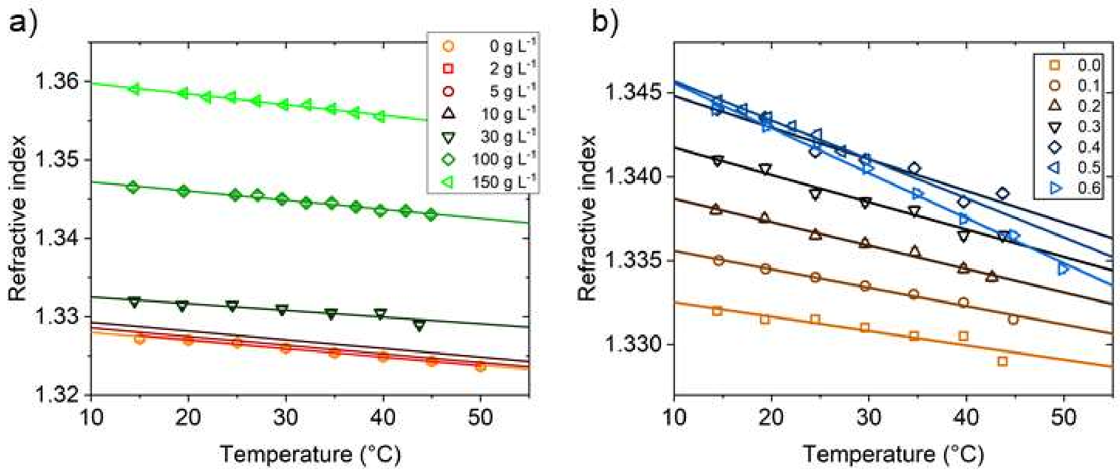

Figure S1. Temperature-dependent refractive indices for (a) PNIPMAM solutions in neat D_2O at the polymer concentrations given in the legend, and (b) for PNIPMAM solutions having a polymer concentration of 30 g L^{-1} in D_2O/CD_3OD mixtures having the CD_3OD volume fractions ϕ_m given in the legend. Symbols: measured values, full lines: linear fits. In (a), the lines for 2-10 g L^{-1} have been interpolated.

S2. Viscosities of the Solvent Mixtures

Temperature-dependent viscosities η of the solvent mixtures are given in Figure S2. The values decrease with temperature and are overall maximum for $\phi_m = 0.4$. The data were fitted using the Vogel-Fulcher-Tammann equation

$$\eta = \eta_0 \exp\left(\frac{B}{T - T_{\mathrm{VF}}}\right) \quad \text{(S1)}$$

with T the absolute temperature and η_0 and T_{VF} being fitting parameters.

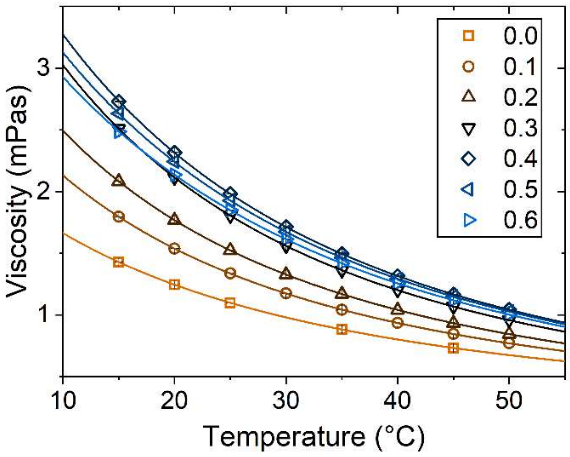

Figure S2. Temperature-dependent viscosities of D_2O/CD_3OD mixtures having the CD_3OD volume fractions ϕ_m given in the legend. Symbols: experimental data, lines: fits of eq S1.

3. Additional Results from DLS and DSC

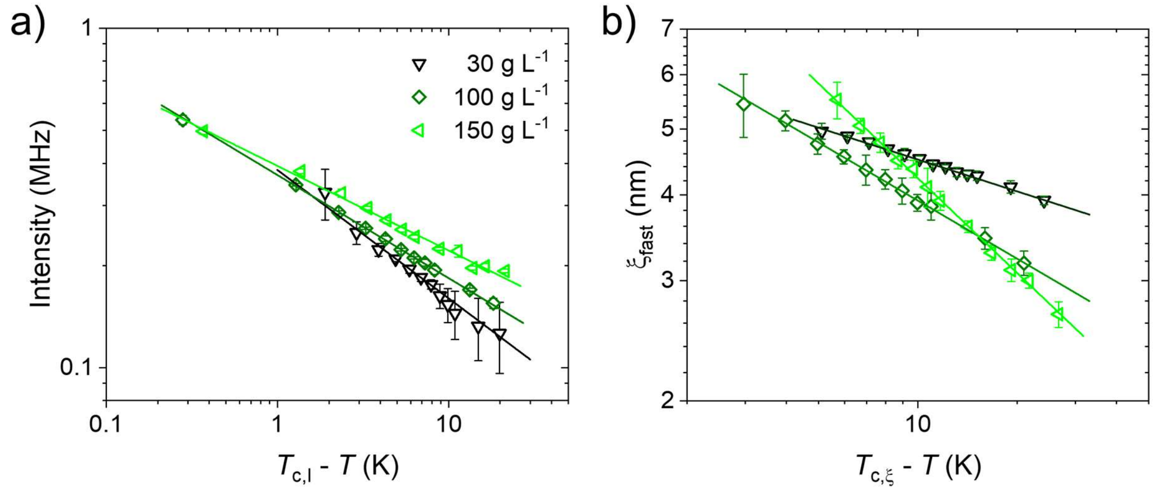

Figure S3. Results from temperature-dependent DLS measurements on PNIPMAM solutions in neat D_2O at the concentrations given in (a), all below the respective T_{cp}. (a) Overall scattered intensities (symbols), plotted in double-logarithmic representation vs. $T_{c,I} - T$. The lines are fits of Eq 6. (b) Correlation lengths of the fast mode, ξ_{fast} (symbols), plotted in double-logarithmic representation vs. $T_{c,\xi} - T$. The lines are fits of Eq 7.

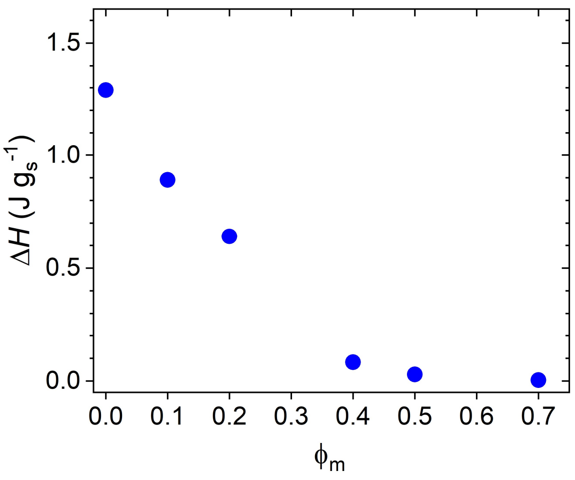

Figure S4. Endothermic enthalpies ΔH, normalized to the mass of the solution, in dependence on the volume fraction of CD_3OD, ϕ_m.

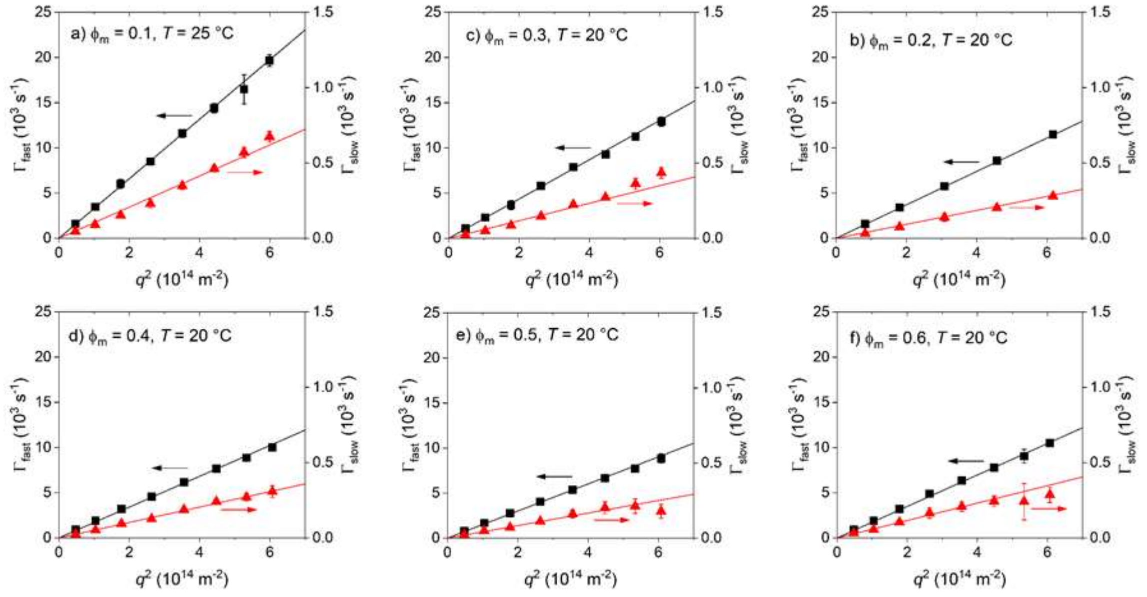

Figure S5. DLS data of the 30 g L^{-1} PNIPMAM solutions in D_2O/CD_3OD at 20 or 25 °C for the volume fractions of CD_3OD, ϕ_m, and temperatures given in the graphs. Relaxation rates Γ_{fast} (black squares, left axis) and Γ_{slow} (red triangles, right axis) in dependence on q^2.

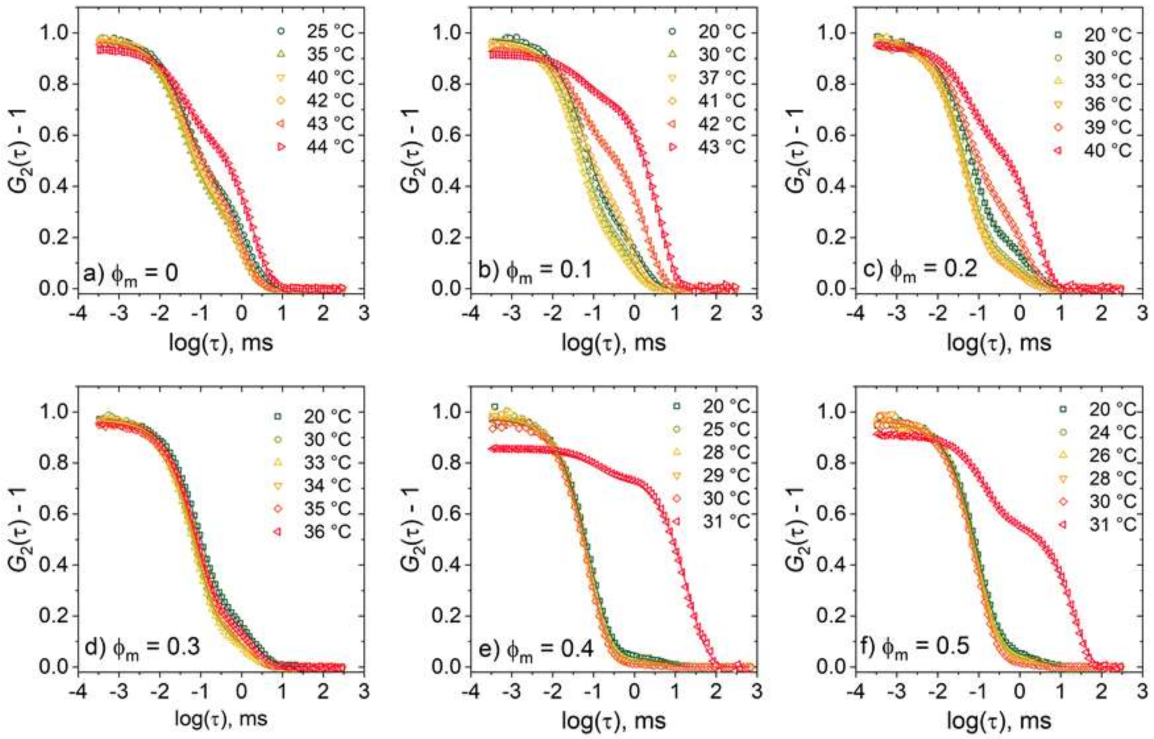

Figure S6. DLS autocorrelation functions of the 30 g L^{-1} PNIPMAM solutions in D_2O/CD_3OD from heating runs for the volume fractions of CD_3OD and temperatures given in the graphs, measured at θ = 90 ° For clarity, only every second experimental data point is shown (symbols). The lines are the fits corresponding to the distributions of relaxation times shown in Figure 8 in the main text.
